# Retraction Note to: Roles of the PI3K/AKT/mTOR signalling pathways in neurodegenerative diseases and tumours

**DOI:** 10.1186/s13578-021-00667-5

**Published:** 2021-08-06

**Authors:** Fei Xu, Lixin Na, Yanfei Li, Linjun Chen

**Affiliations:** 1 Department of Microbiology and Immunology, Shanghai University of Medicine & Health Sciences, 279 Zhouzhu Rd, Shanghai, 201318 China; 2 Collaborative Innovation Center of Shanghai University of Medicine & Health Sciences, Shanghai, 201318 China; 3 Department of Inspection and Quarantine, Shanghai University of Medicine & Health Sciences, Shanghai, 201318 China

## Retraction to: Cell Biosci (2020) 10:54 https://doi.org/10.1186/s13578-020-00416-0

The Editor-in-Chief has retracted this article at the request of Fei Xu. The article contains substantial overlap with another article previously published in Chinese by different authors [1].

All authors agree with this retraction.
